# Efficacy of Preemptive Dexamethasone versus Methylprednisolone in the Management of Postoperative Discomfort and Pain after Mandibular Third Molar Surgery: A Systematic Review and Meta-Analysis

**DOI:** 10.1155/2023/7412026

**Published:** 2023-04-30

**Authors:** Anupam Singh, Kalyana Chakravarthy Pentapati, Murali Venkata Rama Mohan Kodali, Komal Smriti, Vathsala Patil, Gandham Lekha Chowdhary, Srikanth Gadicherla

**Affiliations:** ^1^Department of Oral and Maxillofacial Surgery, Manipal College of Dental Sciences, Manipal, Manipal Academy of Higher Education (MAHE), Karnataka, India; ^2^Department of Public Health Dentistry, Manipal College of Dental Sciences, Manipal, Manipal Academy of Higher Education (MAHE), Karnataka, India; ^3^Department of Oral and Maxillofacial Surgery, College of Dentistry, King Faisal University, Al-Ahsa, Saudi Arabia; ^4^Department of Oral Medicine and Radiology, Manipal College of Dental Sciences, Manipal, Manipal Academy of Higher Education (MAHE), Karnataka, India; ^5^Manipal College of Dental Sciences, Manipal, Manipal Academy of Higher Education (MAHE), Karnataka, India

## Abstract

The corticosteroids have been used for preemptive management of surgical sequelae after mandibular third molar extraction. The aim of this article was to review the efficacy of methylprednisolone versus dexamethasone in the management of postsurgical pain, swelling, and trismus after mandibular third molar surgery. Randomized, double-blinded studies from PubMed, CINAHL, Scopus, DOSS, Cochrane central, and Web of Science were identified by using a search strategy. Randomized controlled trials evaluating the efficacy of use of dexamethasone versus methylprednisolone for mandibular third molar extraction were only considered. The studies involving the use of any other corticosteroid agent were excluded. Outcomes assessed were postoperative pain, the number of rescue analgesics required, swelling, trismus, and adverse events. The search strategy yielded 1046 articles for title and abstract screening, out of which only seven studies were included in the systematic review after full text screening. There was considerable heterogeneity between the studies with regards to the method as well as the parameters assessed. Risk of bias was low in three studies and unclear in other four studies. On pooled analyses, there was no significant difference with respect to pain, rescue analgesics, and swelling in the test and the control group. Forest plot analysis showed that dexamethasone had lesser trismus in early postoperative period (postoperative day 2) as compared to methylprednisolone. None of the included studies reported any adverse effects. Both the corticosteroids have similar efficacy in reducing the postoperative pain and swelling; however, dexamethasone showed statistically significant difference from methylprednisolone in reducing trismus (estimated standardized mean difference of −0.69 mm; 95% CI: −1.01 to −0.38; *p* < 0.0001) in the early postoperative period. However, due to statistical heterogeneity, quality of the evidence for the review was low to moderate. Hence, more studies with larger study sample and low risk of bias are needed to confirm these results.

## 1. Introduction

Transalveolar extraction of an impacted mandibular third molar (M3M) is a routine minor oral surgical procedure. It results in an acute and overt inflammatory response that might lead to postoperative complications such as pain, trismus, and edema [1, 2]. Trismus can lead to functional limitations and edema causing significant esthetic concerns; both of them can potentially affect oral health-related quality of life [3]. Although the effect is for a few days, it is generally unacceptable to the patient and further dissuades them from seeking appropriate treatment. Hence, maxillofacial surgeons attempt to minimize the postoperative sequelae after M3M surgical extraction.

Modification of flap design, atraumatic osteotomy, cryotherapy, and pharmacological agents (corticosteroids) have been tried previously to reduce early postoperative complications [3]. Many maxillofacial surgeons choose a single dose of preemptive corticosteroid during the surgical extraction of M3M. The potent anti-inflammatory action inhibits vasodilatation and decreases cellular exudates and fibrin deposits. The suppression of the vasoactive substances' (prostaglandins and leukotrienes) production reduces the edema [4]. Although the anti-inflammatory action of corticosteroids is well-established, their role in reducing postoperative complications remains inconclusive [5, 6]. The potential adverse effects of the steroids are delayed wound healing and increased risk of infection, usually seen only with prolonged use of corticosteroids. The use of single-dose preemptive corticosteroids has not shown adverse effects [4, 7].

The two most preferred corticosteroids in minor oral surgery are methylprednisolone and dexamethasone. They predominantly exert glucocorticoid action and have minimal effect on sodium retention or mineralocorticoid action. Methylprednisolone is an intermediate-acting corticosteroid with 4-5 times more potency than hydrocortisone. Dexamethasone is a long-acting corticosteroid with 40-50 times more potency than hydrocortisone [8].

A previous systematic review included 28 randomized-controlled trials (RCTs) evaluating the use of corticosteroids in M3M surgery. It was reported that the use of corticosteroids had a significant reduction in postoperative trismus and inflammation. However, there was no consensus regarding the preferred corticosteroid, route, and dosage [9]. A systematic review compared the efficacy of dexamethasone versus methylprednisolone in M3M surgeries, which included RCTs that used the submucosal corticosteroid [10]. With this background, this systematic review evaluated the efficacy of dexamethasone versus methylprednisolone in managing postoperative discomfort and pain after M3M surgery.

## 2. Materials and Methods

### 2.1. Protocol

This systematic review was reported as per the “PRISMA (Preferred Reporting Items for Systematic Reviews and Meta-Analyses)” guidelines [11]. The protocol was registered with the PROSPERO (CRD42020161341).

### 2.2. Inclusion and Exclusion Criteria

The PICO acronym was used to define the research question. The search was conducted for studies on healthy volunteers or asymptomatic patients needing surgical extraction of impacted M3M under local anesthesia. The intervention under study was the administration of preemptive methylprednisolone, which was compared with preemptive dexamethasone. The outcome measures were the postoperative pain assessment using a visual analogue scale (VAS), the number of rescue analgesics consumed, trismus, and edema. Studies of the effect of any other corticosteroid agents and studies conducted for any other surgical procedure other than removal of impacted M3M were excluded.

Trismus assessment is conducted by measuring the change in the maximal interincisal distance (MID) or maximal interincisal opening (MIO) from the preoperative value to the subsequent measurements at follow-up visits after tooth extraction [12]. Postextraction facial swelling assessment described by Ustun et al. involved measurement of three lines, i.e., line joining outer canthus to gonion, tragus to commissure line, and tragus to soft tissue pogonion line [13]. Another method for assessing facial swelling is measuring the “tragus-commissure line”, “gonion-commissure line”, and “gonion-external canthus line” [14]. Alternatively, a 2-line measurement involving gonion-external canthus line and tragus-commissure line has also been described to evaluate facial swelling [15].

Randomized controlled trials published without publication date or language restrictions were included. The laboratory studies, abstracts, case series, review articles, editorials, interviews, discussions, and opinions were excluded.

### 2.3. Search Strategy

**Figure 1:**
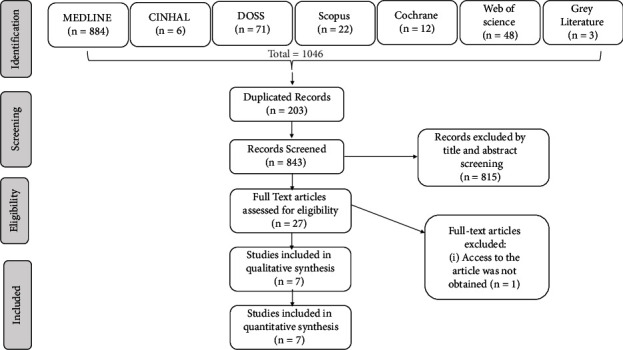
PRISMA flowchart.

Six electronic databases were searched using a combination of terms from inception to June 30, 2022 (Table 1 and Figure 1). In addition, a grey literature search revealed three articles. Also, the references in the included studies were hand searched. The search was carried out using a combination of terms: dexamethasone, methylprednisolone, and M3M (Table 1).

### 2.4. Data Extraction and Management

Two review authors (A.S. and P.K.C.) performed title, abstract, and full-text screening. Two review authors (A.S. and S.G.) independently performed the data extraction. Information extracted was author names, year, sample sizes, mean age, gender distribution, difficulty index, type, dose, and route of corticosteroid administration, and the outcome results such as pain scores, rescue analgesics, swelling, trismus, and complications, if any. Conflicts were resolved after a discussion with the third review author (P.K.C.). A third reviewer (K.S.) resolved conflicts.

### 2.5. Assessment of Risk of Bias in RCTs

The risk of bias was assessed by the tool described in the “Cochrane Handbook for Systematic Reviews of Interventions” [16]. Two independent reviewers (A.S. and S.G.) assessed the risk of bias for the included studies. A third reviewer (P.K.C.) resolved disagreements.

### 2.6. Statistical Analyses

Data analysis was performed using “Review Manager (RevMan)” (Computer program), ver. 5.4.1 (The Cochrane Collaboration, 2020). The heterogeneity of studies was assessed using the *I*^2^ statistic and *χ*^2^. We used the standardized mean difference and random effects model to generate the forest plot.

## 3. Results

### 3.1. Search Results

Six electronic databases PubMed (*n* = 884), CINAHL (*n* = 6), Scopus (*n* = 22), DOSS (*n* = 71), Cochrane central (*n* = 12), and Web of Science (*n* = 48) yielded a total of 1046 articles. After removing duplicated records (203), during abstract and title screening, 815 articles were excluded, and 27 articles were taken up for full-text analysis. Out of 27 articles, permission for 1 article was not obtained, and another article was excluded as it lacked comprehensible data. [15] Finally, seven studies were included for the qualitative and quantitative synthesis (meta-analysis) (Figure 1).

### 3.2. Bias Assessment

Seven studies met the selection criteria according to the “Cochrane collaboration's risk of bias tool” (Table 2). None of the included studies had a low risk of bias across all domains. However, none of the studies had a high risk of bias.

### 3.3. Qualitative Evaluation

Six studies used a preoperative approach [14, 17–21], while the time of steroid administration was not specified in one study [22]. Five studies were conducted on a split-mouth randomized controlled trial design [14, 17–19, 22]. Only one study [17] used different routes of corticosteroid administration, i.e., intravenous administration of methylprednisolone and intramasseteric dexamethasone, while the rest of the studies had similar methods of administration in both methylprednisolone and dexamethasone groups. Two studies used the intramasseteric approach [17, 22], two studies used the submucosal approach [20, 21], and two studies used the oral method of administration of corticosteroids [18, 19]. Six studies used a similar dose of methylprednisolone, i.e., 40 mg; one study [14] used a variable dose of methylprednisolone, i.e., 1.5 mg/kg body weight. Four studies used a 4 mg dose of dexamethasone [14, 17, 21], whereas the other four used an 8 mg dose of dexamethasone [18–20, 22]. Two studies used Pell and Gregory's Class II, Position B type of impacted teeth [18, 19], and four studies specified only similar types of impacted teeth were taken up for trial without specifying the classification category [17, 20–22] and one study [14] did not report on the selection criteria for including the type of impacted teeth in the study.

### 3.4. Quantitative Analysis

#### 3.4.1. Pain Evaluation

**Figure 2:**
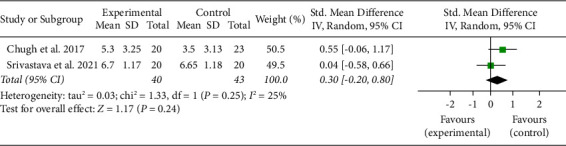
Forest plot for analysis of pain on day 1.

Five studies reported the pain evaluation employing a visual analogue scale (VAS) on a score of 0–10 [18–22], one trial reported pain evaluation by VAS score with calibration of 0–100 [14]. One trial used the number of rescue analgesics used as a means to assess postoperative pain [14]. Pooled analysis for pain scores for day 1 and day 3 was only possible from two studies [20, 22] (Table 3). There were no significant differences in the estimated average standardized mean difference between methylprednisolone and dexamethasone for pain on day 1. There was no significant amount of heterogeneity (Figure 2).

**Figure 3:**
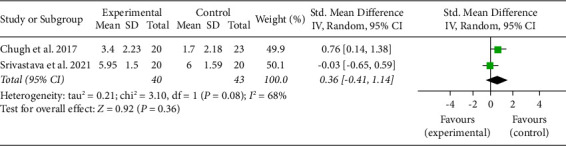
Forest plot for analysis of pain on day 3.

Similarly, there were no significant differences in the estimated average standardized mean difference between methylprednisolone and dexamethasone for the pain on day 3, with substantial heterogeneity (Figure 3).

#### 3.4.2. Trismus Evaluation

In one trial, the absolute maximum mouth opening (MMO) was reported without mentioning the specific unit of measurement. Hence, it could not be considered for analysis [17]. Two studies reported the absolute MMO values for day 2 and day 7. However, no analysis could be conducted due to insufficient data [14, 22]. Four studies evaluated trismus by a change in the MMO from baseline values obtained from preoperative data [18–21] (Table 4).

**Figure 4:**
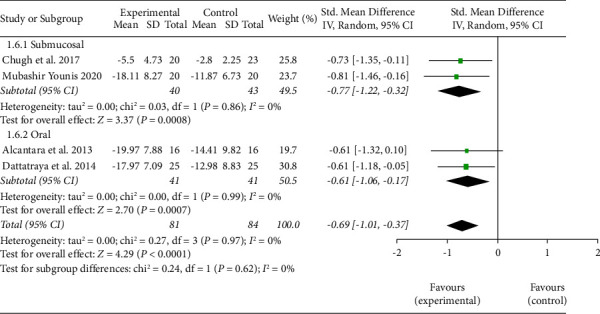
Forest Plot for analysis of trismus on day 2 with subgroup analysis for submucosal and oral route of administration of drugs.

Two studies involved submucosal administration of the drugs [20, 21]. The estimated standardized mean difference for the drugs administered through the submucosal route was −0.77, whereas two studies involved the administration of the drugs orally [18, 19]. The estimated standardized mean difference for the drugs administered orally was −0.61. On cumulative evaluation, the estimated standardized mean difference between two groups for trismus was −0.69 mm (*p* < 0.0001), which favoured dexamethasone on day 2. This difference even though clinically insignificant was found to be statistically significant. There was no heterogeneity among the studies (Figure 4).

**Figure 5:**
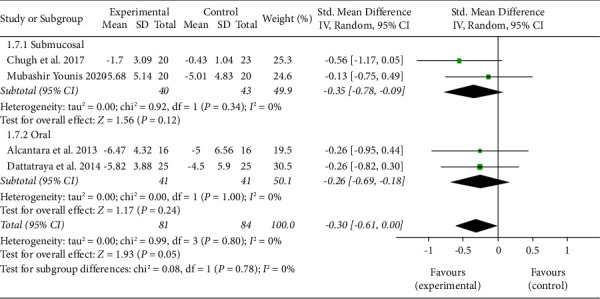
Forest Plot for analysis of trismus on day 7 with subgroup analysis for submucosal and oral route of administration of drugs.

However, no significant difference was seen in the estimated average standardized mean difference between methylprednisolone and dexamethasone on day 7 for trismus on a pooled analysis of all four studies. On subgroup analysis, in the studies involving the submucosal administration of drugs, the estimated standardized mean difference was −0.35. While for the studies involving the oral administration of drugs, the estimated standardized mean difference was −0.26. There was no heterogeneity among the studies (Figure 5).

#### 3.4.3. Swelling Evaluation

Two studies reported the swelling by measurement of two lines, i.e., the canthus-gonion line and tragus-commissure line [20, 21]. Two other studies evaluated swelling by the sum of three lines: Canthus-gonionline + tragus-commissureline + gonion-commissure line [14, 22]. However, due to a lack of data, no analysis was attempted. Three studies evaluated swelling by measurement of three lines described by Ustun et al., i.e., canthus-gonionline + tragus-commissureline + tragus-pogonion line [17–19]. In this, two studies reported the swelling by mean difference from the baseline value [18, 19], while another reported it as a mean value [17] (Table 5). These two studies had a similar standardized mean difference on all follow-ups. Hence, a meta-analysis could not be performed for these outcomes.

#### 3.4.4. Rescue Analgesics

**Figure 6:**
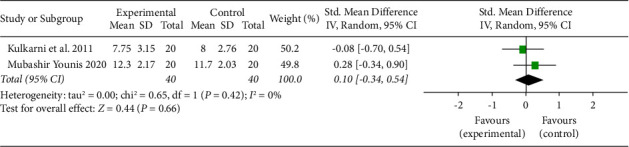
Forest plot for analysis of rescue analgesics.

Only two studies reported rescue analgesics [17, 21] (Table 6). No significant difference in the estimated standardized mean difference between methylprednisolone and dexamethasone concerning the number of rescue analgesics. There was heterogeneity among the studies (Figure 6).

## 4. Discussion

Preemptive administration of corticosteroids for M3M removal surgery effectively reduced postoperative trismus and inflammation [9]. The evidence in support of both preemptive dexamethasone versus methylprednisolone to decrease postsurgical complications of M3M surgery remains equivocal. The previous meta-analysis concluded that dexamethasone was more effective than other oral anti-inflammatory drugs for reducing swelling and trismus after M3M surgery [23]. Another meta-analysis on the evaluation of the effect of submucosal dexamethasone injection as against in M3M surgery suggested that dexamethasone was more effective for reducing postoperative complications such as pain and edema, with no significant effect on trismus [24]. However, another meta-analysis that evaluated the effectiveness of methylprednisolone against placebos in M3M surgery suggested that methylprednisolone significantly reduced pain, edema, and trismus in the early postoperative period [25]. Another meta-analysis evaluating the effectiveness of methylprednisolone against other anti-inflammatory drugs showed that methylprednisolone was significantly better in reducing trismus after 7 postoperative days [26].

We conducted this review to evaluate the effectiveness of dexamethasone versus methylprednisolone in reducing postoperative outcomes after M3M surgery. Ngeow and Lim reported extensively on corticosteroids in M3M surgery [27]. Methylprednisolone has an intermediate duration of action (12–36 h), while dexamethasone is a long-acting corticosteroid (>36 h) [28]. Concerning potency, dexamethasone is more potent, as its 0.75 mg is equivalent to 4 mg dose of methylprednisolone (equivalent to 1 mg of glucocorticoid dose). Dexamethasone has better anti-inflammatory properties (30 x relative to hydrocortisone) than methylprednisolone (5 x relative to hydrocortisone) [29, 30].

Evaluation of facial swelling is a relatively subjective assessment. Following surgery, swelling peaks at 48 hours and has been termed “rebound swelling” [13, 31]. Different methods of assessment of swelling were noted among the included studies with a lack of standardized reporting. These methods varied in the landmarks used for taking the measurements [13, 14, 32]. Owing to different methods of swelling assessment, pooled estimates could not be calculated. Only two studies with a similar method of swelling evaluation and study parameters were used for meta-analysis. Studies by Dattatraya et al. and Alacantara et al. used 40 mg of methylprednisolone and 8 mg of dexamethasone orally. Since dexamethasone is a longer-acting corticosteroid compared to methylprednisolone, it has been reported that it is more effective in reducing postsurgical swelling [14, 18, 19, 23]. In the two studies evaluated for meta-analysis, both studies showed that dexamethasone was more effective in reducing swelling on postoperative days 1 and 2. However, the studies had similar standardized mean differences on all follow-ups, due to which meta-analysis could not be performed.

Trismus following third molar surgery has been attributed to pain and muscle stiffness. The inhibitory feedback on motor cortex excitability from the masseter and lateral pterygoid muscle has also been postulated as one of the possible reasons for trismus [33]. However, this theory remains controversial because of the complex functions of the trigeminothalamic and spinothalamic systems [34]. Irrespective of the mechanism, trismus following third molar surgery remains significant postsurgical sequelae. Corticosteroid exerts an anti-inflammatory effect in the surrounding tissues around the surgical area, which can indirectly reduce the intensity of trismus. Change in the maximal inter-incisal distance from the baseline value noted prior to the procedure was the method followed by four of the studies. The pooled data from these studies showed significantly less trismus in the dexamethasone group in the early postoperative period (POD 2). However, there was no significant difference on the 7th day post-surgery (POD 7). This finding could be explained by the longer half-life of dexamethasone, which would have exerted its effect for a longer time as compared to methylprednisolone.

Prostaglandins and bradykinins are the inflammatory mediators produced at the tissue injury site. These inflammatory mediators are downregulated by the corticosteroid and, hypothetically, should have relieved postsurgical pain. Tissue injury following third molar surgery stimulates neurotransmitters (substance P, glutamate, and calcitonin gene-related peptide) from the nociceptor terminals located in the spinal cord. The corticosteroids do not inhibit these neurotransmitters. Hence, the pain persists, albeit at a lower amplitude, despite the inhibition of the production of the prostanoid [35]. Even though different studies have reported different findings regarding the better agent in pain control, the pooled analysis did not show any significant difference between methylprednisolone and dexamethasone in reducing pain in the postoperative period. Furthermore, the two studies included in the pooled analysis for pain were those conducted by Chugh et al. and Srivastava et al. These two studies used different drug administration modes but used similar doses of steroids.

Apart from using the VAS score, some authors also use the number of rescue analgesics consumed postsurgery to assess pain control [17]. The study protocol could also affect the number of rescue analgesics consumed. Not all the studies specified the postsurgical protocol that the patients followed. Hence, only two studies could be included in the pooled analysis to assess rescue analgesics. In their study, Kulkarni et al. and Younis specified the concept of rescue analgesics as a postoperative pain management protocol. However, the pooled analysis did not lead to any significant results.

Kandamani et al. [10] compared the efficacy of dexamethasone versus methylprednisolone in managing postoperative sequelae after M3M surgery. However, they only evaluated the RCTs involving the submucosal administration of the drugs and included only two studies in their review [15, 20]. In contrast, we included all the RCTs that compared dexamethasone with methylprednisolone in M3M surgeries, irrespective of the route of administration. This is based on the comparative efficacy of two drugs remaining the same if given by a similar route [29]. Another factor that increased the heterogeneity of the studies was the different doses of methylprednisolone and dexamethasone administered across various studies. The complications associated with long-term corticosteroid therapy are well documented, including osteoporosis, infections, obesity, and hyperglycemia [36, 37]. However, complications associated with a single dose of corticosteroids are rare and have been reported in studies involving intralesional injection of corticosteroids. These include the complications such as pain, bleeding, and allergic reaction [38]. In the studies included in the analysis, none of them reported any significant complication which could be attributed to the single dose of corticosteroid.

The findings of this systematic review show that apart from having a significant advantage in reducing the early postoperative trismus (day 2), dexamethasone did not have any other significant difference compared with similarly administered methylprednisolone in reducing postoperative pain and swelling after third molar surgery. However, more studies with large sample sizes and low risk of bias are required to see the effectiveness on pain, trismus, and swelling with standardized assessments and follow-ups along with the inclusion of patient-reported outcomes and complications.

## Figures and Tables

**Table 1:** Search strategies for the databases.

Database	Search strategy
MEDLINE/PubMed	((((dexamethasone[MeSH Terms]) OR (dexamethasone[Title/Abstract])) AND (methylprednisolone[MeSH Terms]) OR (methylprednisolone[Title/Abstract])) AND (third molar[MeSH Terms]) OR (third molar[Title/Abstract]))
SCOPUS	TITLE-ABS-KEY (dexamethasone) OR INDEXTERMS (dexamethasone) AND TITLE-ABS-KEY (methylprednisolone) OR INDEXTERMS (methylprednisolone) AND TITLE-ABS-KEY (third AND molar) OR INDEXTERMS (third AND molar)
CINHAL	TX dexamethasone AND TX methylprednisolone AND TX third molar
DOSS	TX dexamethasone AND TX methylprednisolone AND TX third molar
Web of Science	ALL = (Dexamethasone) AND ALL = (Methylprednisolone) AND ALL = (Third Molar) ALL = (Dexamethasone)
Cochrane	“third molar” in Title Abstract Keyword AND “dexamethasone” in Title Abstract Keyword AND “methylprednisolone” in Title Abstract Keyword

**Table 2:** Risk of bias.

Sr. No.	Author (Year)	Random sequence generation (selection bias)	Allocation concealment (selection bias)	Selective reporting (reporting bias)	Blinding of participants and personnel (performance bias)	Blinding of outcome assessment (detection bias)	Incomplete outcome data (attrition bias)	Other bias if any
1	Loganathan et al. (2011)	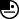	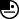	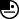	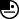	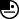	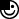	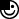
2	Kulkarni et al. (2011)	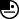	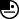	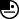	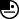	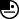	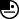	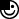
3	Alcantara et al. (2013)	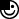	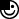	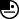	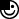	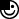	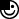	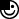
4	Dattatraya et al. (2014)	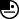	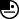	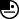	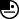	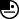	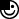	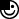
5	Chugh et al. (2017)	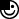	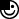	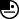	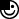	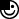	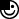	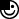
6	Nikhil Srivastava et al. (2021)	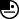	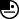	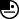	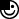	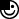	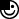	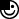
7	Mubashir Younis (2020)	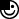	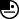	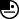	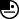	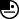	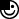	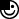

**Table 3:** Pain evaluation.

Author (year)	Method of pain evaluation	Test day 1 mean (SD)	Control day 1 (SD)	Test day 3 (SD)	Control day 3 (SD)	Test day 5 (SD)	Control day 5 (SD)	Test day 7 (SD)	Control day 7 (SD)
Loganathan S et al. (2011)	VAS score (0–100)	9.23 (−)	9.67 (−)	10.67 (−)	11.56 (−)	—	—	—	—
Alcantara et al. (2013)	VAS Score (0–10)^*∗*^	2 (−)	1 (−)	2 (−)	1 (−)	—	—	—	—
Dattatraya et al. (2014)	VAS Score (0–10) – Reported in graphical data	—	—	—	—	—	—	—	—
Chugh et al. (2017)	VAS Score (0–10)	5.3 (3.25)	3.5 (3.13)	3.4 (2.23)	1.7 (2.18)	3.2 (2.65)^#^	1.3 (2.08)^#^	1.5 (1.96)	0.9 (1.99)
Nikhil Srivastava et al. (2021)	VAS Score (0–10)	6.7 (1.17)	6.65 (1.18)	5.95 (1.5)	6 (1.59)	4.17 (1.56)	4.35 (1.56)	3.05 (1.14)$	2.8 (1.05)$
Mubashir Younis (2020)	VAS Score (0–10) – Reported in graphical data	—	—	—	—	—	—	—	—

^*∗*^Median score. ^#^Day 4. ^$^Day 6.

**Table 4:** Trismus evaluation.

Author (year)	Method of maximum mouth opening evaluation	Test day 0 (SD)	Control day 0 (SD)	Test day 2 (SD)	Control day 2 (SD)	Test day 5 (SD)	Control day 5 (SD)	Test day 7 (SD)	Control day 7 (SD)
Loganathan S et al. (2011)	Callipers in mm	44.33 (−)	43.76 (−)	33.33 (−)	32.76 (−)	—	—	40.16 (−)	39.72
Kulkarni et al. (2011)	Interincisal distance	—	—	3.17 (0.36)	2.55 (0.55)	—	—	4.49 (0.41)	4.14 (0.4)
Alcantara et al. (2013)	Reduction in MMO from baseline value in mm	−16.27 (8.13)^*∗*^	−13.83 (8.65)^*∗*^	−19.97 (7.88)	−14.41 (9.82)	−15.59 (6.27)	−13.63 (9.11)	−6.47 (4.32)	−5.00 (6.56)
Dattatraya et al. (2014)	Reduction in MMO from baseline value in mm	−14.64 (7.3)^*∗*^	−12.45 (7.78)^*∗*^	−17.97 (7.09)	−12.98 (8.83)	−14.03 (5.64)	−12.26 (8.19)	−5.82 (3.88)	−4.5 (5.9)
Chugh et al. (2017)	Reduction in MMO from baseline value in mm	—	—	−5.5 (4.73)	−2.8 (2.25)	—	—	−1.7 (3.09)	−0.43 (1.04)
Nikhil Srivastava et al. (2021)	MMO in mm	40.10 (2.47)	39.8 (2.82)	24.25 (3.49)	29.2 (3.75)	—	—	32.95 (3.33)	36.45 (3.82)
Mubashir Younis (2020)	Reduction in MMO from baseline value in mm	—	—	−18.11 (8.27)	−11.87 (6.73)	−17.56 (6.86)^#^	−11.22 (5.42)^#^	−5.68 (5.14)	−5.01 (4.83)

^*∗*^Day 1. ^#^Day 4.

**Table 5:** Swelling evaluation.

Author (year)	Method of evaluation of swelling	Test day 1 (SD)	Control day 1 (SD)	Test day 2 (SD)	Control day 2 (SD)	Test day 3 (SD)	Control day 3 (SD)	Test day 7 (SD)	Control day 7 (SD)
Loganathan et al. (2011)	Sum of 3 lines-canthus-gonion + tragus-commissure + gonion-commissure in mm	299.73^*∗*^ (−)	302.26^*∗*^ (−)	353.69 (−)	314.39 (−)	—	—	302.08 (−)	304.26 (−)
Kulkarni et al. (2011)	Sum of 3 lines—Canthal-gonion + tragus-commissure + tragus-pogonion in mm	—	—	365.9 (22.44)	381.1 (21.73)	—	—	347.05 (21.55)	352 (23.03)
Alcantara et al. (2013)	Sum of 3 lines-Ustun et al.-(Canthus-gonion + tragus-commissure + tragus-pogonion) measured as mean change in base value in mm	5.38 (2.21)	3.31 (2.75)	7.88 (2.36)	4.5 (3.3)	6.13 (2.84)	3.19 (3.29)	1.5 (1.75)	0.25 (0.57)
Dattatraya et al. (2014)	Sum of 3 lines-Ustun et al.-(canthus-gonion + tragus-commissure + tragus-pogonion) measured as mean change in base value in mm	5.91 (2.43)	3.64 (3.03)	8.66 (2.59)	4.95 (3.63)	6.74 (3.12)	3.5 (3.61)	1.65 (1.92)	0.27 (0.62)
Daniel Lim et al. (2015)	Sum of 2 lines—corner of eye to angle and tragus to corner of mouth. Measured as % change in baseline value. Reported in Graphical data only	—	—	—	—	—	—	—	—
Chugh et al. (2017)	Sum of 2 lines—corner of eye to angle and tragus to corner of mouth—measured as mean change in base value in mm			11 (5.24)	5.4 (4.1)			2 (2.98)	1.5 (4.21)
Nikhil Srivastava et al. (2021)	Sum of 3 lines—Tragus-Commissure + Gonion-Commissure + Gonion-external canthus in mm	291.8 (25.78) SD s sum of all three SDs?	291.4 (25.55)	318.6 (30.77)	309.7 (27.37)	—	—	299.25 (25.61)	295.85 (24.48)
Mubashir Younis (2020)	Sum of 2 lines—Tragus-commissure + canthus-gonion. Unit of measurement not defined			9.37 (5.85)	5.25 (4.98)	7.03 (5.01)^#^	3.78 (3.9)^#^	1.12 (2.02)	0.43 (1.99)

^*∗*^Day 0. ^#^Day 4.

**Table 6:** Rescue analgesics.

Author (Year)	Test mean (SD)	Control mean (SD)
Kulkarni et al. (2011)	7.75 (3.15)	80 (2.76)
Mubashir Younis (2020)	12.3 (2.17)	11.7 (2.03)

## Data Availability

The data used to support the findings of this study are available from the studies reviewed in the systematic review.
